# Gene variants in the FTO gene are associated with adiponectin and TNF-alpha levels in gestational diabetes mellitus

**DOI:** 10.1186/s13098-017-0234-0

**Published:** 2017-05-12

**Authors:** Renata Saucedo, Jorge Valencia, Claudia Gutierrez, Lourdes Basurto, Marcelino Hernandez, Edgardo Puello, Guadalupe Rico, Gloria Vega, Arturo Zarate

**Affiliations:** 1 Endocrine Research Unit, National Medical Center, IMSS, Cuauhtemoc 330, 06720 Mexico City, Mexico; 2 Hospital of Gynecology and Obstetrics, Medical Center La Raza, IMSS, Mexico City, Mexico; 3 Unit of Experimental Medicine, UNAM, Mexico City, Mexico

**Keywords:** Adipokines, FTO, Gestational diabetes, Obesity, Single nucleotide polymorphism

## Abstract

**Background:**

Obesity may have a role in the development of gestational diabetes mellitus (GDM). Single-nucleotide-polymorphisms (SNPs) of the FTO (fat mass and obesity associated) gene have been associated with obesity. The aim of this study was to investigate SNPs rs8050136, rs9939609, and rs1421085 of the FTO gene in women with GDM and their associations with maternal pre-pregnancy weight and body mass index, gestational weight gain and mediators of insulin resistance in GDM like leptin, adiponectin, ghrelin and tumor necrosis factor-alpha (TNF-alpha), compared with healthy pregnant controls.

**Methods:**

80 women with GDM and 80 women with normal pregnancy were considered for the present study. Genotyping of selected SNPs in all study subjects was done using the Taq-Man assay and the adipokines and ghrelin were measured by immunoassays. Chi square test, odds ratios (OR) and their respective 95% confidence intervals were used to measure the strength of association between FTO SNPs and GDM.

**Results:**

There was no association among FTO SNPs and GDM. Interestingly, in GDM group, women carrying the risk alleles of the three SNPs had increased TNF-alpha, and decreased adiponectin levels; these associations remained significant after adjusting for pre-gestational body weight and age. Moreover, the risk allele of rs1421085 was also associated with increased weight gain during pregnancy.

**Conclusions:**

The FTP SNPs rs8050136, rs9939609, and rs1421085 are not a major genetic regulator in the etiology of GDM in the studied ethnic group. However, these SNPs were associated with adiponectin and TNF-alpha concentrations in GDM subjects.

## Background

The prevalence of obesity and overweight is increasing around the world [1]. Across the Organisation for Economic Co-operation and Development (OECD) countries, Mexico is the country with the second highest obesity rate among adults and a greater proportion of this population are women [2]. In 2012, among women of childbearing age, 18% were obese [3]. Obesity in women of reproductive age is a known risk factor for numerous health problems, including gestational diabetes mellitus (GDM), pregnancy induced hypertension, preeclampsia, caesarean delivery, post-partum hemorrhage, and delivery of large-for-gestational-age infants [4, 5]. Excessive gestational weight has also been associated with adverse health outcomes for both the mother and her child [6].

Obesity is a neuroendocrine disorder in which both genetic predisposition and environmental factors act in concert [7]. In recent years, genome-wide association studies (GWAS) have identified >90 loci containing single-nucleotide-polymorphisms (SNPs), many in intronic regions, associated with human obesity [8, 9]. The strongest genetic association with risk to polygenic obesity is in SNPs in intron 1 and 2 of the FTO (fat mass and obesity associated) gene [10, 11].

The precise mechanism responsible for FTO’s effect on obesity in humans has been elusive. It has been suggested that the obesity-associated FTO SNPs may affect obesity through greater food intake and increased hunger/lowered satiety and through modulating adiposity factors. The FTO SNP rs9939609 has been found to be significantly associated with levels of circulating ghrelin and leptin [12, 13], which are key mediators of ingestive behavior, and has been significantly associated with serum adiponectin, an antihyperglycemic, antiatherogenic, and anti-inflammatory adipokine [14]. Additionally, FTO SNP rs9939609 has been related to systemic inflammation [15], which is implicated in insulin resistance and diabetogenesis [16].

Increasing evidence suggests that the FTO SNPs influence the expression of genes proximal to FTO in the locus, namely, RPGRIP1L, IRX3, and IRX5 [17–19]. A recent study identified rs1421085 as a causal variant to obesity, which influences the expression of IRX3 and IRX5 in preadipocytes, changing human adipocytes from fuel utilization through enhanced thermogenesis to the function as substrate storage [20].

FTO variants rs9939609 and rs8050136 also confer higher risk for type 2 diabetes (T2D) in an adiposity-dependent manner and have been associated with body mass index (BMI) and GDM in Caucasians women [21–25]. The present study investigates the polymorphisms rs8050136, rs9939609, and rs1421085 of the FTO gene in women with gestational diabetes and their associations with maternal pre-pregnancy weight and BMI, gestational weight gain and mediators of insulin resistance in GDM like leptin, adiponectin, ghrelin and tumor necrosis factor-alpha (TNF-alpha), compared with healthy pregnant controls.

## Methods

### Subjects

We recruited a total of 80 women with GDM and 80 women with normal pregnancy at the Hospital of Gynecology and Obstetrics, Medical Center La Raza, IMSS, Mexico City, Mexico, as described previously [26]. Briefly, the protocol was approved by our institutional review board, and informed consent was obtained from all participants. Gestational diabetes was diagnosed by a 2-h 75 g oral glucose tolerance test at 24–28 weeks of gestation, the cutoff values being >5.2 mmol/L at fasting, >10.0 mmol/L at 1 h, and >7.8 mmol/L at 2 h [27]. All women with GDM began nutritional therapy soon after diagnosis and 40 patients required insulin therapy. Patients with arterial hypertension, renal disease, liver disease, thyroid disorders, or other endocrine or chronic diseases were excluded from this study. In the morning, at gestational week 30 and before initiation of insulin therapy in those women with GDM treated with insulin, anthropometric measurements of height and weight were obtained using a medical scale, and blood samples were taken. Information regarding the demographic features was obtained from all the subjects with the help of a standard questionnaire.

### Assays

The peripheral blood was obtained by a single venipuncture between 7:30 and 8:30 a.m. after an overnight fast. Samples were centrifuged, and serum was separated and frozen at −70 °C until assayed in a single run. Plasma glucose, triglycerides, and total cholesterol were measured by enzymatic assays with a Roche Cobas Mira analyzer using commercial kits (Stanbio Laboratory, Boerne, TX, USA). Insulin and ghrelin concentrations were determined by radioimmunoassay (RIA); insulin was measured using reagents from Siemens Healthcare Diagnostics (Los Angeles, CA, USA), sensitivity was 8.3 pmol/L and intra- and interassay coefficients of variation (CVs) were 5.2 and 7.3%, respectively. Ghrelin was determined using reagents from EMD Millipore Corporation (Billerica, MA, USA); sensitivity was 6.4 pg/mL and CVs were 4.4 and 9.2%. TNF-alpha was measured by chemiluminescent immunoassay (Immulite Analyzer, Diagnostic Products, Los Angeles, CA). Sensitivity was 1.7 pg/mL and CVs were 3.2 and 5.2%.

Genomic DNA was isolated from anticoagulated blood by using the GFX Genomic Blood DNA Purification Kit (AmershamBiosciences) and stored at −70 °C. Genotyping of selected SNPs in all study subjects was done using the Taq-Man assay (Applied Biosystems). The Taq-Man genotyping reaction was performed according to the manufacturer’s protocol [95 °C (10 min), then 40 cycles of 95 C (15 s), and 60 C (1 min)] on an StepOnePlus Real Time PCR system (Applied Biosystems, USA).

BMI was calculated as weight in kilograms divided by the square of height in meters. Insulin sensitivity was assessed by the validated homeostasis model assessment (HOMA) index [28] using the following formula: ${\text{HOMA-IR}} = \left( {{\text{FPG }}*{\text{ FPI}}} \right)/ 2 2. 5,$ where FPG and FPI are fasting plasma glucose (mmol/L) and fasting plasma insulin (μU/mL), respectively.

### Statistical analyses

The Hardy–Weinberg (HW) distribution of the genotypes in the GDM and control groups was assessed. The allele distributions of polymorphisms among patients with GDM patients and normal subjects were evaluated by χ^2^ test and Fisher exact tests accordingly, calculating odds ratios (ORs), 95% confidence intervals (CIs), and corresponding P values. The Kolmogorov–Smirnov statistical test was used to test the normality of the distributions, and a nonparametric statistical analysis was performed. Data were expressed as median (interquartile range 25–75%). The Mann–Whitney U test was used for continuous variables, and the Chi square test was used for categorical data. The associations between genotypes and anthropometric characteristics, adipokines, ghrelin and TNF-alpha in GDM patients were evaluated using analysis of covariance (ANCOVA) with genotypes as a factor and age, pre-gestational body weight, and insulin therapy as covariates. We performed a multiple logistic regression analysis to estimate whether maternal age, maternal BMI, and FTO genotype were suitable as prognostic factors for GDM. P < 0.05 was considered statistically significant. Data were evaluated by using SPSS 21 (SPSS Inc, Chicago, IL).

## Results

Table 1 displays the anthropometric and biochemical characteristics from control and GDM subjects. Age, weight, BMI, glucose, insulin, HOMA-IR and triglycerides were significantly higher in the GDM women compared with controls. GDM patients also had a high prevalence of diabetes in their family history. Gestational weight gain, total cholesterol, leptin, adiponectin, ghrelin and TNF-alpha were not significantly different between groups.

**Table 1:** Anthropometric and biochemical characteristics at the time of recruitment

	Control (n = 80)	GDM (n = 80)	P
Age (years)	24 (18.8–27.3)	33 (28.5–36.0)	<0.001
Pre-pregnancy weight (kg)	55 (49–63)	72 (62.5–79.5)	<0.001
Pre-pregnancy BMI (kg/m^2^)	23 (21–25)	30 (26.7–32.8)	<0.001
Gestational weight gain (kg)	8 (5.6–10)	6.9 (3–10)	0.256
Family history of diabetes (%)	21 (26.0)	52 (65)	<0.001
Fasting glucose (mg/dL)	67.7 (62.8–74.0)	99 (85.8–121.5)	<0.001
Insulin (μIU/mL)	6.7 (4.5–12)	19.2 (6.9–34.9)	<0.001
HOMA-IR	1.09 (0.72–2.0)	4.1 (1.7–10.4)	<0.001
Total cholesterol (mg/dL)	230.4 (214.9–255.3)	237.1 (215.6–268.7)	0.653
Triglycerides (mg/dL)	220.8 (162.1–285.0)	286.6 (225.4–357.1)	<0.001
Leptin (ng/mL)	19.9 (14.3–29.4)	19 (14.0–23.9)	0.902
Adiponectin (ng/mL)	10.8 (7.9–15.2)	8.4 (6.0–10.7)	0.585
Ghrelin (pg/mL)	598.3 (534.4–701.5)	622.4 (505.3–699.8)	0.745
TNF-alpha (pg/mL)	9.5 (7.9–11.7)	10.3 (9.2–12.4)	0.158

Data are expressed as median (interquartile range 25–75%)

*GDM* gestational diabetes mellitus, *BMI* body mass index, *HOMA*-*IR* homeostasis model assessment index

All genotypes in both groups were in HW equilibrium (P > 0.05). We estimated linkage disequilibrium (LD) among the three studied variants: rs8050136 was in complete linkage disequilibrium with rs9939609 and these SNPs and rs1421082 were in a tight LD block (D′ 0.94 and γ^2^ 0.89).

The genotype and allele frequencies of the three studied polymorphisms were not significantly different between the groups (Table 2). As a result of the small number of homozygotes for the rare alleles, we compared the common allele homozygotes and the carriers of the rare allele in a dominant model.

**Table 2:** Genotypic distribution of the studied SNPs in controls and GDM patients

Polymorphisms	Genotype	Control	GDM	OR (95% CI)	P
*FTO rs8050136*	CC	59 (73.8)	61 (76.3)	1.11 (0.54–2.27)	0.855
	CA	20 (25.0)	18 (22.5)		
	AA	1 (1.2)	1 (1.1)		
Risk allele	A				
Risk allele frequency (%)		13.7	12.4		
*FTO rs9939609*	TT	59 (73.8)	61 (76.3)	1.11 (0.54–2.27)	0.855
	TA	20 (25.0)	18 (22.5)		
	AA	1 (1.2)	1 (1.1)		
Risk allele	A				
Risk allele frequency (%)		13.7	12.4		
*FTO rs1421085*	TT	58 (72.5)	64 (80.0)	1.54 (0.75–3.2)	0.278
	TC	20 (25.0)	15 (18.8)		
	CC	2 (2.5)	1 (1.2)		
Risk allele	C				
Risk allele frequency (%)		15.0	10.6		

*SNPs* single nucleotide polymorphisms, *FTO* fat mass and obesity associated gene, *GDM* gestational diabetes mellitus, *OR* odds ratio, *CI* confidence interval

In GDM group, women carrying the A risk allele of rs8050136 and the A risk allele of rs9939609 had increased plasma TNF-alpha, and decreased adiponectin levels; these associations remained significant after adjusting for age and pre-gestational body weight. Moreover, C risk allele of rs1421085 was also associated with both increased weight gain during pregnancy and with TNF-alpha (Table 3). In contrast, no significant associations of FTO rs8050136, rs9939609, and rs1421085 were found with any of the studied biomarkers analyzed in control group (data not shown).

**Table 3:** Association of FTO SNPs with anthropometric characteristics, adipokines, ghrelin and TNF-α in GDM patients

*FTO rs8050136/ rs9939609*	CC/ TT	CA and AA/ TA and AA	P value	P corr
Pre-pregnancy weight (kg)	72 (60–79)	70.5 (63.5–79.6)	0.729	0.804
Pre-pregnancy BMI (kg/m^2^)	30 (26.9–33.2)	29.8 (26.3–32.1)	0.805	0.901
Gestational weight gain (kg)	6.3 (2.8–10.0)	8.5 (3.6–12.5)	0.283	0.352
Fasting glucose (mg/dL)	101.5 (88.7–123.6)	90.4 (76.1–119.5)	0.313	0.319
Insulin (μIU/mL)	19.2 (7.5–32.8)	17.3 (4.4–35.8)	0.951	0.986
HOMA-IR	4.1 (1.9–10.2)	4.0 (4.0–10.9)	0.671	0.772
Leptin (ng/mL)	19.9 (15.8–26.9)	15.5 (13.4–23.1)	0.211	0.169
Adiponectin (ng/mL)	10.1 (7.3–11.2)	5.5 (3.8–7.8)	0.043	0.048
Ghrelin (pg/mL)	626.1 (487.7–728.8)	618.7 (505.9–690.3)	0.864	0.654
TNF-alpha (pg/mL)	10.0 (9.0–11.9)	12.8 (10.1–13.2)	0.040	0.043

*FTO rs1421085*	TT	TC and CC		
Pre-pregnancy weight (kg)	74 (63.5–80)	70.0 (62.5–82.9)	0.894	0.902
Pre-pregnancy BMI (kg/m^2^)	30.7 (27.0–34.0)	28.6 (25.8–32.2)	0.583	0.632
Gestational weight gain (kg)	6.0 (2.0–10.0)	9.0 (5.5–13.5)	0.004	0.012
Fasting glucose (mg/dL)	100.2 (87.9–123.5)	90.9 (75.6–119.9)	0.651	0.775
Insulin (μIU/mL)	19.5 (7.9–36.8)	16.6 (4.2–40.6)	0.846	0.942
HOMA-IR	5.2 (2.1–11.5)	4.0 (0.86–11.7)	0.898	0.875
Leptin (ng/mL)	20.6 (16.5–24.5)	14.7 (13.3–22.4)	0.462	0.563
Adiponectin (ng/mL)	9.7 (6.8–11.2)	6.6 (4.3–9.0)	0.101	0.223
Ghrelin (pg/mL)	626.1 (489.9–735.4)	574.0 (494.2–638.6)	0.419	0.532
TNF-alpha (pg/mL)	9.8 (9.0–11.7)	12.0 (10.0–13.0)	0.048	0.05

Data are expressed as median (interquartile range 25–75%)

*SNPs* single nucleotide polymorphisms, *GDM* gestational diabetes mellitus, *FTO* fat mass and obesity associated gene, *BMI* body mass index, *HOMA*-*IR* homeostasis model assessment index

P corr, adjusted for age and pre-gestational body weight

In the logistic regression analysis, the association between GDM and maternal age as well as weight was significant (OR 1.24, 95% CI 1.15–1.35, and P = 0.001; OR 1.08, 95% CI 1.05–1.12, and P = 0.001, respectively).

## Discussion

GDM accounts for one of the most frequent gestational complications, affecting 4–18% of all pregnancies worldwide, depending on the GDM criteria used [29]. In recent decades, the number of patients with GDM has increased significantly; the main factors for this rise are the increasing prevalence of overweight and obesity in both developed and developing countries [30, 31]. Likewise, some other risk factors for GDM include age, ethnic origin and a family history of T2D [32]. In this study, GDM patients were reported to be associated with older age, higher BMI and greater parental history of T2D, when compared to healthier pregnant women.

Additionally, evidence is accumulating that susceptibility to GDM has a genetic component [25, 33]. FTO has been reported to be associated with obesity, and recent studies have shown the association of some FTO SNPs with GDM, like rs8050136 or rs9939609 [23–25]; however, to our knowledge, no studies on rs1421085 have been previously reported. In the present study, we examined the association of these genetic variants in FTO gene in women with GDM.

We found no association between the FTO SNPs rs8050136 and rs9939609 with GDM, in accordance with Cho et al. and Fabrico et al. [34, 35]. However, our results differed from three studies that examined rs9939609, which demonstrated an association among the A-alleles and the risk for GDM [23–25]. We hypothesized that the A-alleles’ low frequency added to the small sample size of this study could be the major factors for the divergence of our results. It has been suggested that the effects of all three SNPs in the FTO gene may be apparent only in populations with higher minor allele frequency [36]. The frequencies of the rare A-alleles observed for the GDM group were lower than those reported for Europeans and Euro-Brazilians [23–25, 35]. On the other hand, these frequencies are similar to those reported for Korean women [34]. To date, most of the genetic association studies have been performed in white populations.

Several genetic variants of FTO have been related to body weight, hip and waist circumference and BMI in numerous populations of European origin [37–39]. Nevertheless, we did not find any association between the FTO SNPs and the pre-pregnancy anthropometric characteristics analyzed. Our results differed from those of Lawlor et al. and Gaillard et al. which demonstrated an association among the risk variants in rs9939609, and rs8050136 and pre-pregnancy weight and BMI [40, 41]. However, we observed that the risk allele C in SNP rs1421085 was associated with greater gestational weight gain in GDM group. Nevertheless, given the composite nature of gestational weight gain, further examination of this outcome might be undertaken by examining the association of this variant with maternal fat stores and fetal adiposity. It should be pointed out that this variant was identified recently as a causal variant in preadipocytes, where the risk allele affects the basic anabolic function of the adipocyte, shifting it from substrate storage to fuel dissipation through increased mitochondrial uncoupling [20].

The genetic variant rs9939609 evaluated in the present study has been involved in appetite ratings through regulation of circulating ghrelin and leptin [12, 13]. Our data did not show these associations; however, we measured total ghrelin, which includes both acylated and desacylated forms. Measuring the acylated form of this hormone, might have been an advantage if we consider that ghrelin is biologically active only in the acylated form [42]. Regarding leptin, the association of FTO polymorphisms with this adipokine appears to be mediated via increased adiposity, as in some studies the association disappears when correcting by BMI [43, 44]. In our study, we did not find an association of FTO polymorphisms with BMI.

In addition, considerable data support a role for adipocyte-derived cytokines, such as adiponectin, and TNF-alpha in the pathogenesis of GDM [45–47]. Therefore, we undertook to investigate the influence of FTO genetic variants on maternal adipokines. In the present study, no statistical differences were found at these hormone levels between GDM and controls. However, interestingly higher levels of pro-inflammatory TNF-alpha and lower levels of adiponectin were associated with the FTO rs8050136 and rs9939609 risk allele A in GDM group. Both associations remained significant after adjusting for age, maternal pre-gestational body weight and insulin therapy. Our results are mainly consistent with those of De Luis et al., who also observed that the minor A allele of the FTO rs9939609 was significantly associated with lower serum adiponectin concentrations independently of potential confounders including adiposity [14]. Regarding the association of the FTO SNPs with systemic inflammation, a German study has shown that rs9939609 A-allele was associated with increased levels of C-reactive protein (CRP) [15]. These findings were significant, independent of the adiposity level. However, a recent study by Zimmermann et al. in a population of Danish men representing a broad range of BMI did not find an independent association between the obesity associated FTO rs9939609 variant and systemic inflammation [43]. To our knowledge no previous studies have investigated the relationship between FTO SNPs and TNF-alpha, only one study to date has examined the association between FTO mRNA expression and expression of TNF-alpha in subcutaneous adipose tissue (SAT), and found that SAT FTO mRNA expression was related to SAT TNF-alpha expression, linking adipose tissue FTO expression and a proinflammatory molecule implicated in insulin resistance [48]. Several studies have suggested that increased levels of TNF-alpha may be related to the development of GDM independent of BMI [49]. TNF-alpha impairs insulin signalling by increasing serine phosphorylation of insulin receptor substrate (IRS)-1, and diminishing insulin receptor (IR) tyrosine kinase activity [50].

This study is subject to certain limitations, the first being the differences in age and weight between women with GDM and women with normal pregnancy. A control group consisting of age- and BMI-matched pregnant women without GDM might have been more suitable. Second, our study was underpowered to detect associations of FTO SNPs with GDM, probably because of their low frequencies. Finally, for adiposity, we only considered BMI, a surrogate for body fatness, and body fat percentage is a more accurate measurement.

## Conclusions

The present study suggests that the FTP SNPs rs8050136, rs9939609, and rs1421085 are not a major genetic regulator in the etiology of GDM in the studied ethnic group. However, these SNPs were associated with serum adiponectin and TNF-alpha concentrations in GDM subjects. Additional larger studies are required to examine the association of FTO genetic variants in Mexican women.

## Abbreviations

GDM: gestational diabetes mellitus
SNPs: single-nucleotide-polymorphisms
FTO: fat mass and obesity associated
BMI: body mass index
TNF-alpha: tumor necrosis factor-alpha
OR: odds ratio
OECD: Organisation for Economic Co-operation and Development
GWAS: genome-wide association studies
DNA: deoxyribonucleic acid
PCR: polymerase chain reaction
HOMA-IR: homeostasis model assessment index
CI: confidence intervals
ANCOVA: analysis of covariance
HW: Hardy–Weinberg
LD: linkage disequilibrium
T2D: type 2 diabetes
CRP: C-reactive protein
SAT: subcutaneous adipose tissue
IRS-1: insulin receptor substrate
IR: insulin receptor
